# Long‐Term Single‐Molecule Tracking in Living Cells using Weak‐Affinity Protein Labeling

**DOI:** 10.1002/anie.202413117

**Published:** 2024-11-25

**Authors:** Claudia Catapano, Marina S. Dietz, Julian Kompa, Soohyen Jang, Petra Freund, Kai Johnsson, Mike Heilemann

**Affiliations:** ^1^ Institute of Physical and Theoretical Chemistry Johann Wolfgang Goethe-University Frankfurt Max-von-Laue-Str. 7 60438 Frankfurt Germany; ^2^ Department of Chemical Biology Max Planck Institute for Medical Research Jahnstr. 29 69120 Heidelberg Germany; ^3^ Institute of Physical and Theoretical Chemistry IMPRS on Cellular Biophysics Max-von-Laue-Str. 7 60438 Frankfurt Germany

**Keywords:** single-molecule fluorescence, single-particle tracking, self-labeling protein tags, weak-affinity binders, membrane receptors

## Abstract

Single‐particle tracking (SPT) has become a powerful tool to monitor the dynamics of membrane proteins in living cells. However, permanent labeling strategies for SPT suffer from photobleaching as a major limitation, restricting observation times, and obstructing the study of long‐term cellular processes within single living cells. Here, we use exchangeable HaloTag Ligands (xHTLs) as an easy‐to‐apply labeling approach for live‐cell SPT and demonstrate extended observation times of individual living cells of up to 30 minutes. Using the xHTL/HaloTag7 labeling system, we measure the ligand‐induced activation kinetics of the epidermal growth factor receptor (EGFR) in single living cells. We generate spatial maps of receptor diffusion in cells, report non‐uniform distributions of receptor mobility, and the formation of spatially confined ‘hot spots’ of EGFR activation. Furthermore, we measured the mobility of an ER‐luminal protein in living cells and found diffusion coefficients that correlated with the ER nano‐structure. This approach represents a general strategy to monitor protein mobility in a functional context and for extended observation times in single living cells.

## Graphical abstract

A self‐labeling protein tag in combination with exchangeable fluorophore ligands bypasses photobleaching and enables long‐term single‐molecule imaging in living cells. This extends observation times in single cells and enables direct monitoring of ligand‐induced receptor activation in living cells.

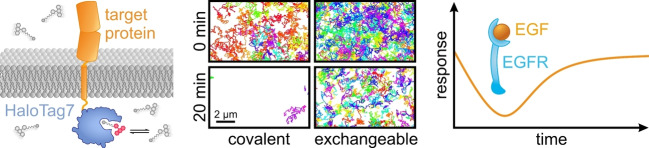

## Introduction

The plasma membrane of a cell constitutes a dynamic interface between the intra‐ and extracellular space and serves as a gate for information exchange. Embedded within this lipid bilayer are membrane receptors, the key elements of this communication, that respond to ligand binding by forming multi‐protein complexes and initiate a signaling cascade inside the cell.[^1^, ^2^] Membrane receptor activation involves intricate interactions with other membrane proteins and lipids. Thus, functional characterization requires methods that allow the visualization of living cells.

Single‐particle tracking (SPT) has developed into a powerful tool to measure the mobility and interactions of single proteins in living cells.[^3^, ^4^, ^5^, ^6^, ^7^] In an SPT experiment, the movement of fluorophore‐labeled single proteins is measured, and mobility trajectories are generated, providing spatiotemporally‐resolved information on diffusion properties.^[8]^ A variety of methods for fluorophore labeling was established, including fluorescent proteins,^[9]^ fluorophore‐labeled ligands,[^10^, ^11^, ^12^] or nanobodies,[^13^, ^14^] as well as self‐labeling protein tags such as HaloTag[^15^, ^16^, ^17^] or SNAP‐tag.[^18^, ^19^] The integration of photoactivatable fluorescent proteins allowed SPT at high protein densities and allowed for high‐density mapping of protein mobility.^[20]^

As a fluorescence method, SPT is challenged by photobleaching, which affects both the lifetime of trajectories as well as the total observation time for a single cell.^[3]^ The trajectory lifetime, defined by the maximum observation time of a single molecule, is mainly limited due to the photon budget that is available from a single fluorophore. More photostable fluorescent probes, such as quantum dots, provide longer trajectories, yet might pose a challenge because of their size and more challenging conjugation chemistry.^[21]^ The total observation time of a single cell labeled with high‐affinity or covalent fluorescent labels is limited by photobleaching of all labels, typically to a time span of seconds to a few minutes.[^7^, ^22^, ^23^, ^24^, ^25^] This can be bypassed by using weak‐affinity fluorophore labels, which continuously bind to and unbind from a protein target, from a reservoir.^[26]^ For example, retro‐engineered HaloTags designed for reversible substrate exchange enabled longer observation times.^[27]^ Recently, the implementation of DNA as a protein label, conjugated to protein‐targeting nanobodies, was shown to enable enhanced observation times for both single trajectories and single cells.[^28^, ^29^]

Diffusion properties can serve as a proxy readout for membrane protein activation. As ligand‐induced activation of membrane receptors occurs at the time scale of several minutes,[^30^, ^31^] SPT experiments monitoring membrane receptor activation were so far conducted by sequential single‐molecule imaging of many single cells.[^13^, ^32^]

Here, we aimed to extend the observation time in single cells through continuous monitoring of protein mobility. For this purpose, we used the self‐labeling protein tag HaloTag7 (HT7) in combination with recently developed exchangeable HaloTag Ligands (xHTLs).^[33]^ We demonstrate long‐term SPT in single living cells and largely independent of photobleaching. This allowed us to conduct long‐term experiments in living cells: as a first example, we monitored the ligand‐induced activation of the membrane receptor epidermal growth factor receptor (EGFR) in the same single cell; as a second example, we measured the diffusion properties of an ER‐luminal protein and correlated this to the ER nano‐structure.

## Results and Discussion

**Figure 1:**
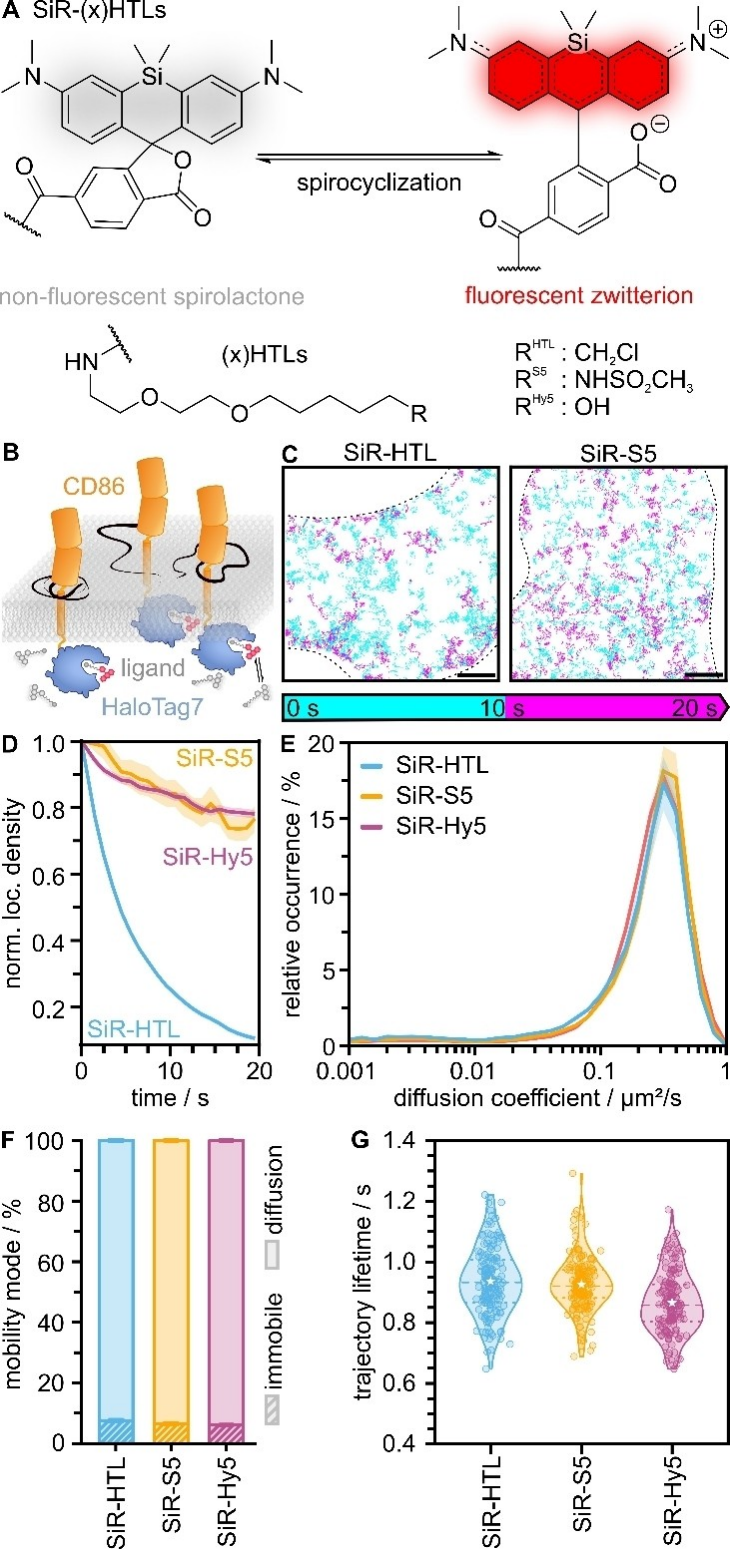
Single‐particle tracking in living cells using exchangeable HaloTag Ligands (xHTLs). (A) Chemical structures of the reversible equilibrium between the non‐fluorescent spirocyclic state and fluorescent zwitterionic state of SiR and different SiR‐derivatives of (x)HTLs. (B) Principle of xHTL‐SPT shown for the transmembrane protein CD86‐HT7. Fluorogenic xHTLs reversibly bind to and unbind from the HT7 tag fused to the intracellular region of CD86 and enable single‐molecule tracking. (C) Single‐molecule trajectories of exemplary cells acquired using the covalent SiR‐HTL (1 nM) or xHTL SiR‐S5 (1 nM) as probes on CD86‐HT7. Trajectories are color‐coded based on their appearance in the first (cyan) or latter half (magenta) of the 20 s acquisition time. Scale bar 5 μm. (D) Mean number of localizations per area binned into 1 s intervals, normalized to the respective data in the first frame, and plotted over time for HT7/SiR‐HTL (blue), HT7/SiR‐S5 (yellow), and dHT7/SiR‐Hy5 (magenta). (E) Relative occurrence of the mean diffusion coefficient per cell for SiR‐tagged xHTLs and the covalent SiR‐HTL. (F) Percentage of mobility modes per cell for SiR‐HTL, SiR‐S5, and SiR‐Hy5 (1 nM each). Single‐molecule trajectories were assigned to the classes immobile or diffusion. (G) Lifetime of single‐molecule trajectories of the covalent SiR‐HTL and exchangeable SiR‐S5 binding to HT7 and SiR‐Hy5 binding to dHT7. Dashed lines represent the median, stars the mean, and dotted lines the interquartile range. All errors represent the standard error of the mean (SEM). 160 cells were acquired for each condition in (D)–(G).

The self‐labeling protein tag HT7 in combination with exchangeable HaloTag Ligands that repetitively bind to and unbind from a protein target was shown to bypass photobleaching in various live‐cell fluorescence microscopy applications.[^33^, ^34^, ^35^] We, therefore, reasoned that this labeling strategy should enable long observation times in SPT experiments in single living cells. xHTLs comprise of a rhodamine dye (or its carbo and silicon rhodamine analogs) and a PEG_2_‐C_5_ alkane sulfonamide ligand (S5), specifically targeting the probe to the HT7 protein (Figure 1A, Figure S1A). Rhodamine dyes have been engineered towards fluorogenicity by exploiting their reversible spirocyclization equilibrium between a fluorescent zwitterion and a non‐fluorescent, but highly cell‐permeable spirolactone .[^36^, ^37^] Upon binding to the HT7 surface, the fluorescent form is stabilized, which leads to a signal increase. The modest fluorogenicity of the used xHTLs may further enhance the signal‐to‐background.^[33]^ However, while fluorogenicity can be a useful feature, it is not essential for obtaining high‐quality SPT data where low probe concentrations are used.

To evaluate this concept, we fused HT7 to the intracellular domain of CD86, a monomeric membrane protein,^[38]^ generated U‐2 OS cell lines expressing CD86‐HT7 and targeted the protein tag with two different silicon rhodamine (SiR) (x)HTLs (Figure 1AB, Figure S1A). We measured the mobility of single CD86‐HT7 proteins in living cells and generated single‐molecule trajectories. We then compared the occurrence of single‐molecule trajectories in living cells for a non‐covalent xHTL (SiR‐S5) to a covalent HTL (SiR‐HTL) and found a strong decrease for the covalent SiR‐HTL already after 10 s in contrast to SiR‐S5 (Figure 1CD). The dead‐mutant dHT7 (D106A), an orthogonal protein tag to HT7,^[33]^ was labeled with xHTL SiR‐Hy5 and showed very similar preservation of the fluorescence signal over time (Figure 1D). Non‐specific binding of all tested HT7 ligands to the plasma membrane was low in control experiments in U‐2 OS wild‐type cells that do not express a HT7 fusion protein (Figure S2).

To evaluate the xHTL‐HT7/‐dHT7 labeling concept for diffusion analysis, we compared the diffusion coefficient and mode of exchangeably labeled CD86 fusions to covalently labeled CD86‐HT7. Independent of the HaloTag‐ligand combination used, the distribution of diffusion coefficients as well as the immobile fraction were similar, yielding mean diffusion coefficients of approx. 0.3 μm^2^/s and about 7 % immobile particles (Figure 1EF, Table S1, Table S2). We next determined the trajectory lifetime of SiR‐labeled ligands and found reasonably similar values of 0.94±0.03 s (SiR‐HTL), 0.93±0.04 s (SiR‐S5) and 0.87±0.02 s (SiR‐Hy5) (Figure 1G, Table S3).

We next evaluated the influence of the fluorophore on the diffusion analysis. For this purpose, we used xHTLs conjugated to the highly fluorogenic rhodamine dye JF_585_, measured the diffusion properties of CD86‐HT7/‐dHT7, and found very similar diffusion coefficients of approximately 0.3 μm^2^/s (Figure S1B, Table S1). We found some small variations in the fraction of immobile particles for JF_585_‐S5 and JF_585_‐Hy4, as compared to SiR‐xHTLs (Figure S1C, Table S2). These results indicate that diffusion coefficients and diffusion modes are largely independent of the fluorophores tested and the type of the ligand.

The trajectory lifetime in an SPT experiment determines the accuracy of the diffusion analysis through the mean‐squared displacement (MSD).[^39^, ^40^] For exchangeable labels such as xHTLs, it depends on i) its binding time to the HT7/dHT7 protein, ii) fluorophore photobleaching, and iii) potential photodamage of the HT7/dHT7 protein. SiR‐S5, SiR‐Hy5 and SiR‐HTL show similar trajectory lifetimes of 0.87–0.94 s (Figure 1G, Table S3). In comparison, binding times of xHTLs were reported around 2 s (SiR‐S5) and 1 s (SiR‐Hy5).^[33]^ This indicates that the trajectory lifetime of SiR‐S5 is more determined by fluorophore photobleaching or photodamage of the protein tag, while the trajectory lifetime of SiR‐Hy5 is also influenced by a shorter binding time to dHT7. The trajectory lifetime of JF_585_‐xHTLs was found to be shorter (0.6 s) than for SiR‐labeled xHTLs (Figure S1D, Table S3). This indicates that the major contribution here is fluorophore photobleaching. The fluorescence signal of JF_585_‐xHTLs over time remained almost constant, indicating a continuous exchange at the tag (Figure S1E, Table S3). Overall the trajectory lifetimes of xHTLs determined in this work are similar to that of fluorophore‐labeled nanobodies or ligands.[^13^, ^14^, ^41^]

We next fused HT7 or dHT7 to the intracellular domain of CTLA‐4, a dimeric membrane protein,^[42]^ and performed SPT experiments in live U‐2 OS cells stably expressing the fusion protein. Again, we observed only a minor decrease of the fluorescence signal over time, and diffusion properties of CTLA‐4 fusions were found to be similar for a variety of xHTL‐fluorophore/HT7/dHT7 combinations (Figure S3). Taken together, the xHTL‐HT7/‐dHT7 system in combination with SPT experiments provides robust information on protein diffusion, while bringing in the additional benefit of minimal signal loss over time.

We observed that xHTL labeling generates a homogeneous distribution of single‐molecule trajectories in cells, whereas HTL labeling leads to more trajectories at the cell border (Figure 1C), a phenomenon often seen in SPT experiments.[^11^, ^32^, ^43^, ^44^] To quantify this observation, we measured the fluorescence signal in living cells at the cell border and compared it to the remaining cell surface area (Figure S4). For the exchangeable probe SiR‐S5, the signal was constant throughout the cell surface area, whereas for the covalent ligand SiR‐HTL, the fluorescence signal was increased at the cell border, indicating a higher protein concentration. We attribute this increase in fluorescence intensity at the cell border to photobleaching of covalent HTLs in the field of imaging of the basal membrane, and replenishment of SiR‐HTL labeled HT7through diffusion from the apical membrane across the cell borders (Figure S4).

**Figure 2:**
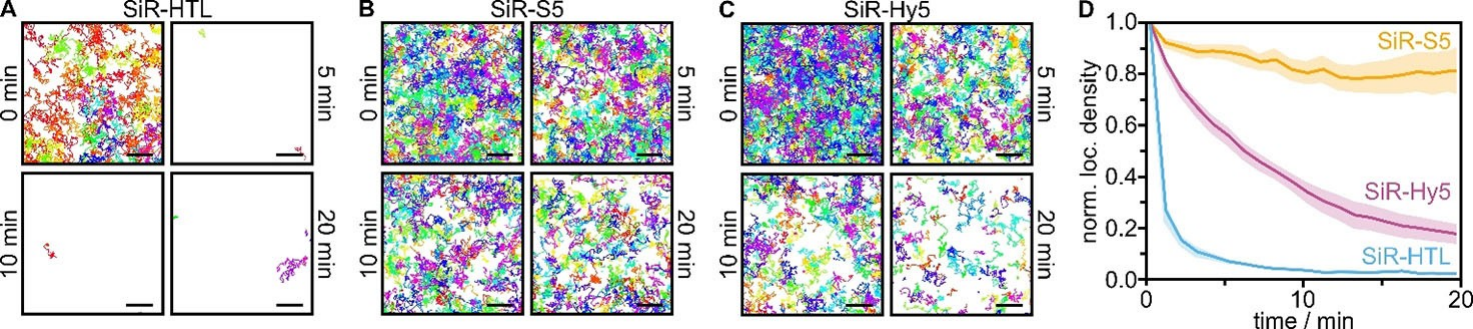
Long‐term single‐particle tracking of CD86‐HT7/dHT7 in living U‐2 OS cells. (A, B, C) Single‐molecule trajectories of CD86 in exemplary cells obtained from (A) HT7/SiR‐HTL (covalent), (B) HT7/SiR‐S5, or (C) dHT7/SiR‐Hy5 (exchangeable). Probe concentration was 1 nM. For each snapshot, the trajectories occurring within 1 min are shown (scale bars 2 μm). (D) Mean number of localizations per area binned into 1 min intervals, normalized to the respective data in the first frame, and plotted over time for CD86‐HT7 or CD86‐dHT7 imaged with SiR‐HTL (blue, N=5 cells), SiR‐S5 (yellow, N=5 cells), and SiR‐Hy5 (magenta, N=6 cells) (1 nM ligand concentration). Error bands represent the SEM.

Motivated by the preservation of fluorescence signal and trajectory density, we attempted to establish long observation times in single living cells by using xHTL/HT7 for repetitive and continuous labeling of target proteins. We conducted long‐term SPT experiments of HT7/dHT7‐tagged CD86 in living cells for up to 20 min using the xHTLs SiR‐S5 and SiR‐Hy5. We found that CD86‐HT7 labeled with the exchangeable SiR‐S5 yielded a constant number of trajectories per time in living cells, whereas, with the covalent SiR‐HTL, the density dropped off quickly (Figure 2AB). This finding is corroborated by the single‐molecule events detected over time, which quickly decrease for the covalent SiR‐HTL, and remain at a higher level (SiR‐Hy5) or even almost constant (SiR‐S5) for the exchangeable ligands (Figure 2, Figure S3E). These findings qualify xHTLs for long‐term SPT experiments in living cells and spatial mapping of the diffusion landscape.

Receptor mobility can serve as a readout to monitor functional states. In cells stimulated with a receptor‐targeting ligand, a slower diffusion, or immobilization, can be correlated with activation.^[45]^ Since the activation occurs on a timescale of several minutes,^[30]^ the accessible observation time of single‐cell SPT experiments was so far a limitation to monitor receptor activation in individual cells. This has been bypassed by sequential imaging of several cells and temporal tiling of SPT data.[^13^, ^32^] We reasoned that xHTL/HT7 labeling should provide a direct solution to monitor receptor activation.

**Figure 3:**
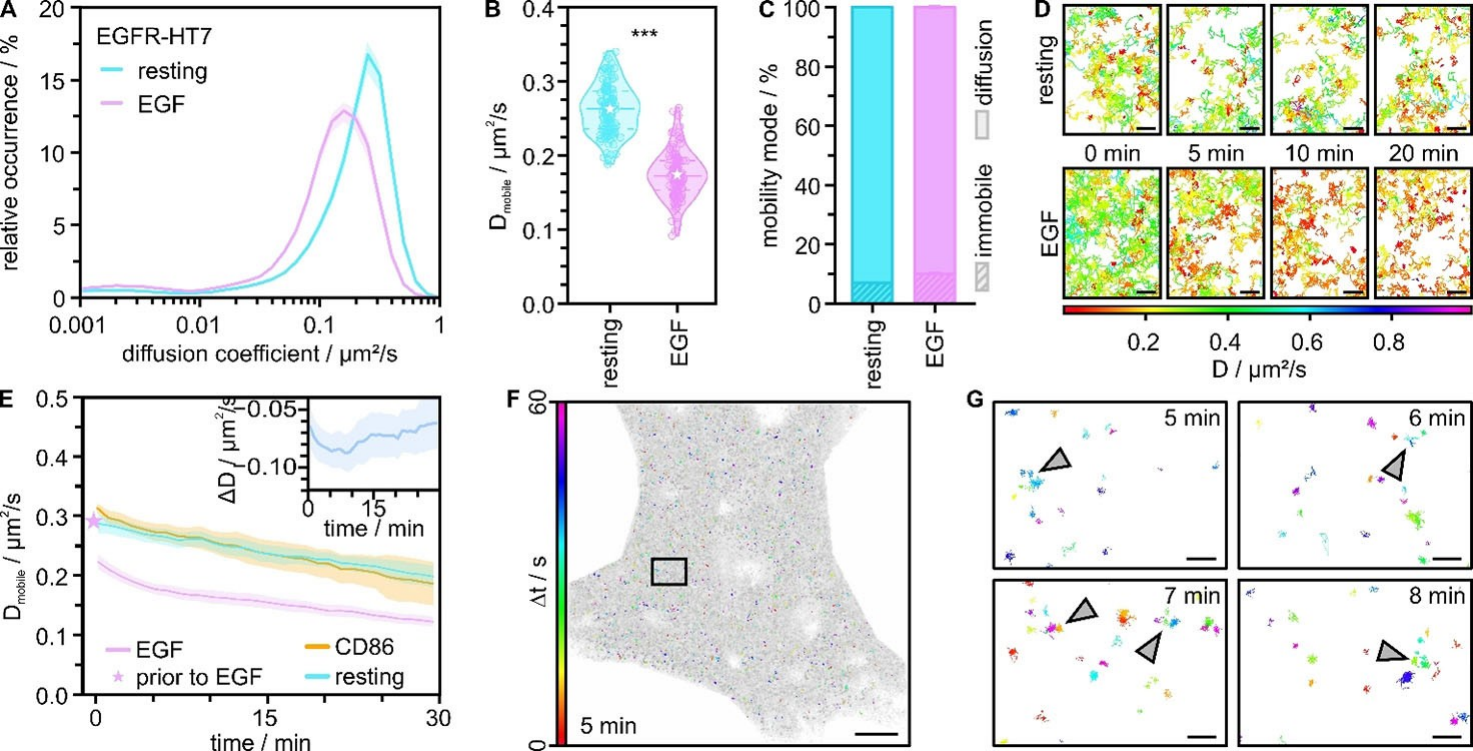
Long‐term single‐particle tracking of transiently expressed EGFR‐HT7 in living U‐2 OS cells measured with 1 nM SiR‐S5. (A) Relative occurrence of the mean diffusion coefficient per cell for resting (cyan) and EGF‐treated (20 nM) cells (magenta). (B) Distribution of diffusion coefficients of mobile EGFR‐HT7 extracted from resting and EGF‐treated cells. Dashed lines represent the median, stars the mean, and dotted lines the interquartile range. P<0.001 (***) indicates highly significant difference between means. (C) Percentage of mobility modes per cell for EGFR in unstimulated and EGF‐stimulated cells. Single‐molecule trajectories were assigned to the classes immobile or diffusion. Data in A−C were taken from 20 s measurements (N=120 cells). (D) Single‐molecule trajectories detected at different time points of a long‐term measurement for resting and EGF‐stimulated cells color‐coded by the assigned diffusion coefficient (scale bar 1 μm). (E) Mean diffusion coefficient of mobile trajectories binned into 1 min intervals and plotted over time for CD86‐HT7 labeled with SiR‐S5 (orange, N=4 cells), EGFR‐HT7 imaged with SiR‐S5 in resting (cyan) (N=5 cells), and EGF‐treated cells (magenta) (N=4 cells). Stars represent the diffusion coefficient in cells before EGF stimulation. The inlay shows the difference plot for the diffusion coefficient of EGFR‐HT7 in EGF‐treated cells. All errors represent the standard error. (F) All trajectories detected over the 30 min acquisition time (gray) overlaid by the immobile trajectories from the period of 300–360 s (color‐coded by their time of appearance within one minute, rainbow) (scale bar 5 μm). (G) Zoom‐in of the box in (F) at different time points (5, 6, 7, and 8 min) of the measurement. Immobile trajectories are clustering in local areas (indicated by arrowheads). Trajectories are color‐coded by their time of appearance within one minute (scale bar 500 nm).

Therefore, we measured the diffusion properties of the transiently expressed EGFR before and after stimulation with its native ligand EGF. EGFR is a membrane receptor that has been extensively studied with various SPT methods.^[46]^ We fused HT7 to the C‐terminal (intracellular) site of EGFR, labeled it with SiR‐S5, and conducted SPT experiments in single living U‐2 OS cells. First, we calculated the diffusion coefficient and mode of EGFR from 20 s measurements in resting and EGF‐stimulated cells. We observed a reduction in the diffusion coefficient (from 0.245±0.004 μm^2^/s to 0.158±0.003 μm^2^/s) and an increase in immobile EGFRupon EGF treatment (from 6.9±0.2 % to 10.1±0.4 %) (Figure 3ABC, Figure S5, Table S2). These findings are in line with previously published work reporting diffusion coefficients in a range of 0.02–0.5 μm^2^/s or 0.005–0.29 μm^2^/s in unstimulated or EGF‐stimulated cells, respectively.[^22^, ^25^, ^45^, ^47^, ^48^, ^49^, ^50^, ^51^, ^52^, ^53^] The decrease of the diffusion coefficient in EGF‐stimulated cells is attributed to EGFR dimerization and activation.^[54]^

We next performed SPT experiments of EGFR‐HT7 in living cells over an extended observation time of 30 min and analyzed its diffusion properties. We quantified the diffusion coefficient of mobile EGFR‐HT7 over time in untreated cells and EGF‐treated cells and compared it to CD86‐HT7 (Figure 3DE). We found that EGFR‐HT7 and CD86‐HT7 show a continuous decrease of the diffusion coefficient over 30 min, which might be a result of minor phototoxic effects (Figure 3E).^[55]^ However, the decrease of the diffusion coefficient was much stronger for EGFR‐HT7 in EGF‐treated cells, particularly in the first 10 min. To extract the activation kinetics of EGFR, we used the diffusion profile of untreated EGFR‐HT7 as a baseline and subtracted it from the diffusion profile of EGFR‐HT7 in EGF‐treated cells. This delivered a difference plot for the diffusion coefficient of untreated and EGF‐stimulated EGFR‐HT7, which showed a dip at around 5–10 min, followed by a recovery (Figure 3E, inset). This corresponds to the activation kinetics of EGFR following EGF treatment measured directly in a single living cell, matching reported cell biology data.[^30^, ^31^]

Our method also allowed us to spatially map membrane sites where EGFR activation occurs (Figure 3FG, Figure S6). We found a non‐uniform distribution of immobile EGFR‐HT7 at different time points, and the appearance of local ‘hot spots’ of sub‐micrometer size that accumulated several receptors at short distances for a short time (Figure 3G, arrowheads). Such ‘hot spots’ indicate the presence of membrane regions where signaling hubs build preferentially and endocytosis through clathrin‐coated pits might occur.[^45^, ^56^] Similarly sized ‘hot spots’ were found in diffraction‐limited microscopy in living cells.^[50]^ A non‐uniform distribution of receptor activation was also reported for the EGFR/HER2 heterodimer.^[57]^

**Figure 4:**
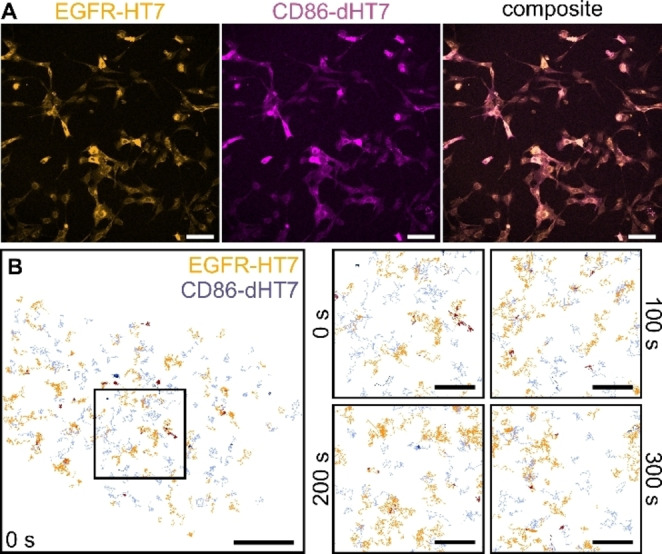
Dual‐color SPT of two orthogonal HaloTags. (A) Confocal microscopy images of U‐2 OS cells transiently expressing EGFR‐HT7 labeled with 100 nM SiR‐S5 (orange) and CD86‐dHT7 labeled with 100 nM JF_585_‐Hy4 (magenta) together with the composite of both channels (scale bars 100 μm). Nearly all transfected cells express both fusion proteins. (B) Single‐molecule trajectories reconstructed from 20 s of an SPT experiment for EGFR‐HT7 (orange) and CD86‐dHT7 (purple) labeled with 1 nM SiR‐S5 and JF_585_‐Hy4, respectively (scale bar 5 μm). Mobile trajectories are represented in light colors, immobile trajectories in dark colors. The box marks the region shown as zoom‐ins at different time points on the right (scale bars 2 μm).

We next demonstrated long‐term dual‐color SPT, profiting from the two orthogonal HaloTag variants, which were previously used as orthogonal labels in two‐color super‐resolution microscopy.^[33]^ EGFR‐HT7 and CD86‐dHT7 were co‐transfected into U‐2 OS cells (Figure 4A) and simultaneously tracked in living cells using the two orthogonal exchangeable ligands SiR‐S5 and JF_585_‐Hy4 (Figure 4B).

**Figure 5:**
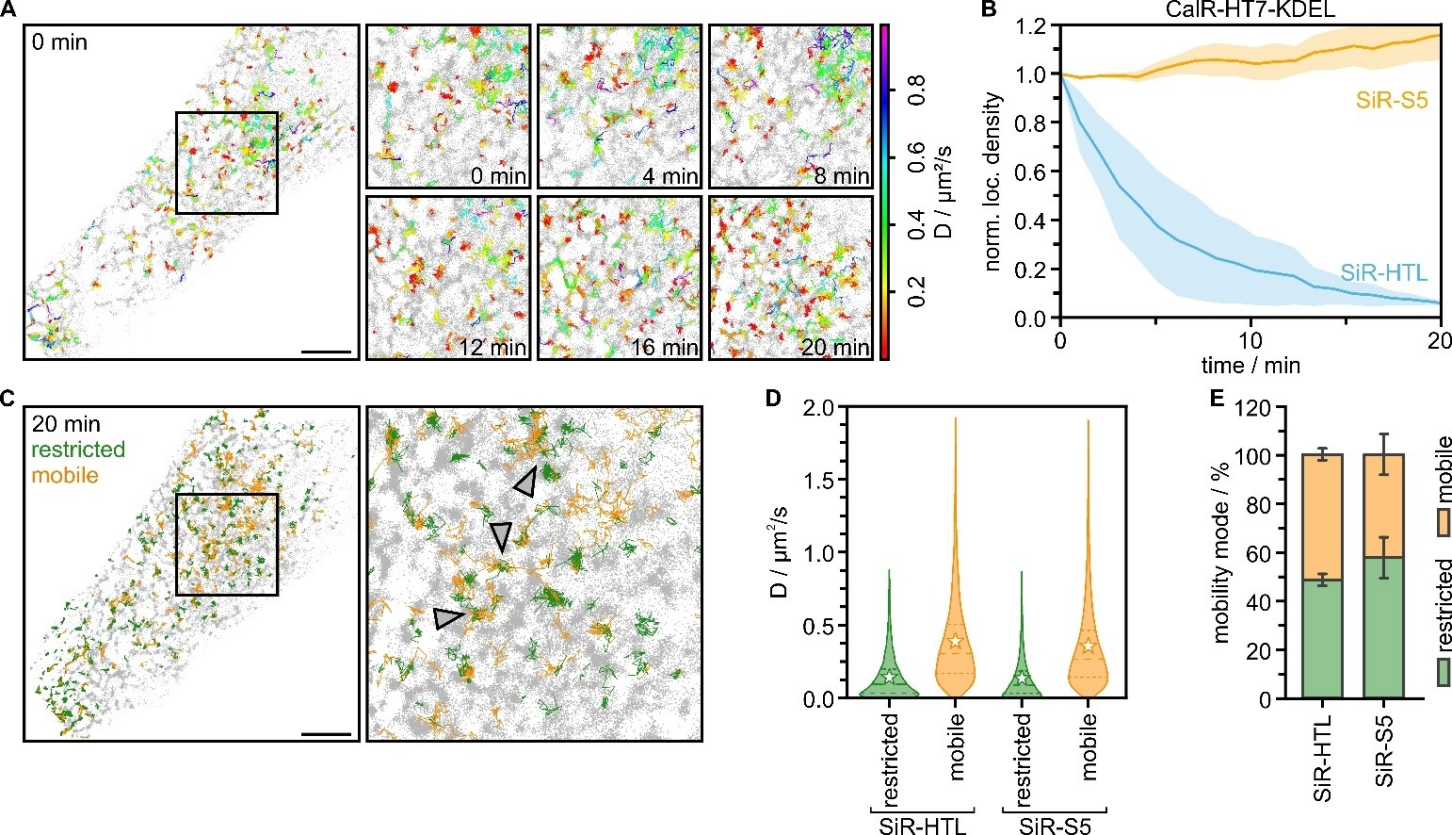
Long‐term single‐particle tracking of calreticulin (CalR)‐HT7‐KDEL in living U‐2 OS cells measured with the exchangeable ligand SiR‐S5 or the covalent ligand SiR‐HTL. (A) Single‐molecule trajectories (rainbow, color‐coded by their diffusion coefficient) of SiR‐S5 (0.5 nM) detected over a time of 1 min at different time points (0, 4, 8, 12, 16, and 20 min) overlayed over the detected localizations (grey) in the same time window. (B) Mean number of localizations per area binned into 1 min intervals, normalized to the respective data in the first frame, and plotted over time for CalR‐HT7‐KDEL imaged with SiR‐HTL (blue, N=2 cells) and SiR‐S5 (yellow, N=3 cells) (0.5 nM ligand concentration). (C) Single‐molecule trajectories of the same cell as in (A) after 20 min measurement time, color‐coded by their modes of motion. Restricted movement is colored in green and mobile diffusion in orange. Restricted movement is predominantly located in the ER nodes (indicated by arrowheads) in comparison to free movement along the ER tubules. (D) Distribution of diffusion coefficients from each trajectory of CalR‐HT7‐KDEL classified as restricted or mobile for measurements using SiR‐HTL and SiR‐S5. Dashed lines represent the median, stars the mean, and dotted lines the interquartile range. (E) Percentage of mobility modes (restricted and mobile) per cell for CalR‐HT7‐KDEL. All errors represent the SEM. Scale bars are 5 μm, zoom‐ins are 10×10 μm in size.

To extend this approach, we conducted SPT experiments of an intracellular protein, by targeting HT7 to the lumen of the endoplasmic reticulum (ER) by fusing it to a calreticulin (CalR)‐KDEL sequence (Figure 5A). We recorded single‐protein trajectories for long acquisition times (Figure 5ABC) and found diffusion coefficients similar to previously reported values (Figure 5D, Table S4).^[58]^ We observed an accumulation of restricted, slower diffusion (0.14±0.04 μm^2^/s) at ER nodes and faster diffusion (0.36±0.04 μm^2^/s) along ER tubules, in line with previous reports.^[58]^ This demonstrates the applicability of our approach for long‐term tracking of intracellular proteins.

## Conclusion

We introduce the self‐labeling protein tags HaloTag7 and dHaloTag7 in combination with weak‐affinity and exchangeable fluorophore ligands for long‐term SPT in single living cells. The extended observation time enables SPT experiments in single cells for several tens of minutes. This facilitates the observation of cellular processes such as receptor activation in the same individual living cell. The xHTL/HT7 labeling pair profits from the availability of engineered cell lines and HT7‐fusion proteins and is not limited to extracellular target sites.

In summary, this method has the potential to become a valuable tool for studying long‐term protein mobility in individual living cells with single‐molecule resolution.

## Supporting Information

The authors have cited additional references within the Supporting Information.[^59^, ^60^, ^61^, ^62^, ^63^, ^64^, ^65^, ^66^]

## Conflict of Interests

J. K. and K. J. are listed as inventors on a patent application related to the exchangeable HaloTag ligands, and filed by the Max Planck Society. Abberior GmbH Göttingen commercializes the exchangeable HaloTag ligands. All other authors declare no competing interests.

1

## Supporting information

As a service to our authors and readers, this journal provides supporting information supplied by the authors. Such materials are peer reviewed and may be re‐organized for online delivery, but are not copy‐edited or typeset. Technical support issues arising from supporting information (other than missing files) should be addressed to the authors.

Supporting Information

## Data Availability

The datasets generated in this study are available in the EMBL BioImage Archive, https://www.ebi.ac.uk/biostudies/bioimages/studies/S‐BIAD1369.
