# Supplementary material for: Long‐Term Single‐Molecule Tracking in Living Cells using Weak‐Affinity Protein Labeling
Source: Angew Chem Int Ed Engl. 2024 Nov 25;64(1):e202413117. doi: 10.1002/anie.202413117 (PMC11701368; doi:10.1002/anie.202413117)
Supplement: Supplementary file 1 — Supporting Information [file ANIE-64-e202413117-s001.pdf]

Angewandte Chemie
Eine Zeitschrift der Gesellschaft Deutscher Chemiker

Supporting Information

Long-Term Single-Molecule Tracking in Living Cells using Weak-Affinity Protein Labeling

*C. Catapano, M. S. Dietz, J. Kompa, S. Jang, P. Freund, K. Johnsson, M. Heilemann**

Supporting Information

Long-time single-molecule tracking in living cells using weak-affinity protein labeling

Claudia Catapano*[1] & Marina S. Dietz*[1], Julian Kompa[2], Soohyen Jang[1,3], Petra Freund[1], Kai Johnsson[2], Mike Heilemann[1,3,#]

*These authors equally contributed to this work.

[1]Institute of Physical and Theoretical Chemistry, Goethe-University Frankfurt, Frankfurt, Germany

[2]Department of Chemical Biology, Max Planck Institute for Medical Research, Heidelberg, Germany

[3]IMPRS on Cellular Biophysics, Frankfurt am Main, Germany

[#]Corresponding author, contact: heilemann@chemie.uni-frankfurt.de

Table of Contents

Experimental Procedures 2

Fluorescent probes 2

Plasmids 2

Generation of stable cell lines 2

Cell culture 2

Sample preparation 3

Live-cell microscopy 3

Confocal microscopy 4

Data analysis 4

Supporting Figures 6

Supporting Tables 10

References 11

Experimental Procedures

Fluorescent probes

All fluorescent xHTLs (SiR-S5, SiR-Hy5, JF_{585}-S5, and JF_{585}-Hy4) were prepared as described in Kompa *et al.*[33] SiR-HTL was kindly provided by B. Réssy and synthesized according to the literature procedure.[36]

Plasmids

HaloTag7 (HT7) or the dead-mutant of the HaloTag7 (dHT7) was genetically fused to the C-terminus of CD86 (Addgene plasmid #98284),[59] CTLA-4 (Addgene plasmid #98285)[59] and EGFR (Addgene plasmid #32751)[60] by replacing the fluorescent protein (mEos2 or EGFP) by molecular cloning (Gibson assembly[61]). To target HaloTag to the endoplasmic reticulum, a TOM20-dHaloTag7-T2A-CalR-HaloTag7-KDEL construct was used.[33] For expression in mammalian cells, the fusion construct was subcloned into a pcDNA5/FRT/TO vector (Invitrogen, Thermo Fisher Scientific, Waltham, MA, USA). All plasmid sequences were verified by Sanger sequencing (Microsynth AG, Balgach, Switzerland).

Generation of stable cell lines

Stable cell lines were generated using the Flp-IN T-REx™ system (Invitrogen). In brief, U-2 OS Flp-In T-REx cells[62] were co-transfected with a 1:10 ratio of pcDNA5-FRT-TO-GOI and pOG44 (Invitrogen) plasmids using the Lipofectamine3000 reagents (Invitrogen) according to the manufacturer’s protocol. The next day, cells were selected with 100 µg/mL hygromycin B (Gibco, Thermo Fisher Scientific, Waltham, MA, USA) for 2 d. After recovery, the cells were treated with 100 µg/mL doxycycline (Sigma-Aldrich, St. Louis, MO, USA) for 24 h to induce protein production previous to fluorescence-assisted cell-sorting (FACS) on a Melody™ Cell sorter (BD Biosciences, Franklin Lakes, NJ, USA) for transgene expression.

Cell culture

U-2 OS wild-type cells (CLS Cell Lines Service GmbH, Eppelheim, Germany) and all stable cell lines were cultivated in growth medium (Dulbecco's Modified Eagle Medium: Nutrient Mixture F12 without phenol red (Gibco) supplemented with 1% GlutaMAX (Gibco), 10% FBS (Sartorius, Göttingen, Germany), 100 U/mL penicillin (Gibco), and 100 µg/mL streptomycin (Gibco)) at 37 °C and 5% CO_2 in an automatic CO_2 incubator (Model C150, Binder GmbH, Tuttlingen, Germany).

For live-cell microscopy, cells were seeded to a density of 4×10^4 cells per sample onto PLL-PEG-RGD-functionalized coverslips in 6-well plates as described elsewhere.[12] After 2 d incubation, protein expression was induced with 100 ng/mL doxycycline (Sigma-Aldrich). Cells were incubated for a further day before performing live-cell microscopy experiments.

For live-cell imaging of EGFR-HT7, the respective plasmid was transiently transfected into U-2 OS wild-type cells. 35×10^4 cells were seeded in 6-well plates, transfected 2 d later with 1 µg/well plasmid DNA using Lipofectamin LTX (Invitrogen) according to the manufacturer’s protocol, and incubated at 37 °C and 5% CO_2. After 6 h, cells were transferred onto PLL-PEG-RGD-coated coverslips via trypsinization and split 1:3. The transferred cells were incubated for 1 d on the coated coverslips in growth medium at 37 °C and 5% CO_2 before imaging.

For confocal and two-color live-cell imaging of EGFR-HT7 and CD86-dHT7, the respective plasmids were transiently co-transfected into U-2 OS wild-type cells. 30×10^4 cells were seeded in 6-well plates, transfected after 2 d with 1 µg/well per plasmid using jetOPTIMUS transfection reagent (Polyplus, Illkirch, France) according to the manufacturer's protocol, and incubated at 37 °C and 5% CO_2. After 7 h, cells were transferred onto PLL-PEG-RGD-coated coverslips via trypsinization and split 1:2. The transferred cells were incubated for 1 d on the coated coverslips in growth medium at 37 °C and 5% CO_2 before imaging.

Sample preparation

For live-cell imaging using covalently binding HaloTag Ligands (HTLs), cells were stained with 1 nM SiR-HTL to label membrane proteins and with 0.5 nM SiR-HTL to label the endoplasmic reticulum in growth medium for 30 min at 37 °C and 5% CO_2 prior to experiments. Then, coverslips were installed into custom-built holders and rinsed three times with FluoroBrite DMEM (Gibco), also used as imaging medium. For live-cell imaging with exchangeable HaloTag Ligands (xHTLs), coverslips were directly mounted into the holders, covered with FluoroBrite DMEM as imaging medium, and supplemented with 1 nM ligand (SiR-S5, SiR-Hy5, JF_{585}-S5 or JF_{585}-Hy4, introduced in Kompa *et al.*[33]) for imaging of membrane proteins or 0.5 nM SiR-S5 for imaging of the endoplasmic reticulum. Samples were mounted into a stagetop incubator (Okolab, Otaviano, Italy) at 25 °C for 10 min before measurements.

For EGF-stimulated samples, the ligand (PeproTech, Thermo Fisher Scientific, Waltham, MA, USA) was added to the sample directly on the microscope to a final concentration of 20 nM (see below).

Live-cell microscopy

Single-color live-cell microscopy experiments were conducted at 25 °C on a commercial widefield microscope (N-STORM; Nikon, Düsseldorf, Germany). The system was controlled via NIS Elements (v4.30.02, Nikon), and LCControl (Agilent, Santa Clara, California, USA), and equipped with an oil-immersion objective (100×Apo TIRF oil, NA 1.49), a stage top incubator (Okolab, Pozzuoli, Italy) and an EMCCD camera (Andor iXon, DU-897U-CS0-BV, Andor, Belfast, UK), operated in total internal reflection fluorescence (TIRF) mode to image target proteins in the membrane, or in highly inclined and laminated optical sheet (HILO) mode to image the endoplasmic reticulum. Samples were illuminated by a 561 nm at 0.5 kW/cm^2 or 647 nm laser at 0.4 kW/cm^2. For long-term measurements of EGFR, a reduced 647 nm laser power of 0.06 kW/cm^2 was used. The following camera parameters were set: EM gain 200, pre-amplifier gain 3, read-out rate 17 MHz, and active frame transfer. Image stacks of 256×256 px with a pixel size of 157 nm were recorded with µManager (v1.4.22)[63] at an integration time of 20 ms. Either 1,000 (standard measurements), 60,000 (long-term measurements of CD86, CTLA-4, and CalR-HT7-KDEL), or 90,000 frames (long-term measurements of EGFR) were recorded per cell, while each sample was imaged for a maximum of 30 min. For long-term measurements of EGF-stimulated samples, a cell was recorded for 1,000 frames in resting condition, then 20 nM EGF were added, and the same cell was imaged for 90,000 frames.

Two-color live-cell microscopy experiments were performed on a home-built Olympus IX-71 inverted TIRF microscope (Olympus Deutschland GmbH, Hamburg, Germany) equipped

with a nosepiece stage (IX2-NPS, Olympus Deutschland GmbH) to provide z-plane adjustment and minimization of drift during measurements. Two lasers (561 nm, 200 mW Sapphire and 637 nm, 140 mW OBIS, both Coherent Inc., Santa Clara, CA, USA), colinearly superimposed using a dichroic mirror (H 568 LPXR superflat, AHF Analysentechnik AG, Tübingen, Germany), served as excitation sources. The sample was illuminated with both lasers after passing an acousto-optical tunable filter (AOTF; AOTFnC-400.650-TN, AA Opto-Electronic, Orsay, France). The two lasers were coupled by a fiber collimator (PAF-X-7-A, Thorlabs, Dachau, Germany) into a single-mode optical fiber (P5-460AR-2, Thorlabs) and subsequently re-collimated to a diameter of 2 mm (60FC-0-RGBV11-47, Schäfter & Kirchhoff, Hamburg, Germany). The collinear beams were directed to a 2-axis galvo scanner mirror system (GVS012/M, Thorlabs) where electronic steering, controlled by an in-house Python script, allowed switching between illumination modes. The excitation light passed two telescope lenses (AC255-050-A-ML and AC508-100-A-ML, Thorlabs) focusing them onto the back focal plane of the objective (UPlanXApo, 100x, NA 1.45, Olympus Deutschland GmbH). In a filter cube, which directs the beam into the objective, two clean-up and rejection bandpass filters together with a dichroic mirror were installed (Dual Line Clean-up ZET561/640x, Dual Line rejection band ZET 561/640, Dual Line beam splitter zt561/640rpc, AHF Analysentechnik AG). Fluorescence light was collected through the same objective and passed the dichroic mirror toward the detection path. An Optosplit II (Cairn Research Ltd, UK) split the emission light around 643 nm into two channels with a beam splitter and two bandpass filters (H643 LPXR, 605/52 BrightLine HC, 679/41 BrightLine HC, AHF Analysentechnik AG). The spatially separated SiR and JF_{585} channels were simultaneously detected on an EMCCD camera (iXon Ultra X-10971, Andor Technology Ltd, Belfast, UK). JF_{585} and SiR were excited with 0.1 kW/cm^2 (637 nm) or 0.05 kW/cm^2 (561 nm), respectively, in circular TIRF mode. The following camera parameters were set: EM gain 200, pre-amplifier gain 3, read-out rate 17 MHz, and active frame transfer. Image stacks of 256×256 px with a pixel size of 159 nm were recorded with µManager (v2.0.0)[63] at an integration time of 20 ms.
For each sample, 10 background measurements with 1,000 frames were taken in areas without cells.

Confocal microscopy

Samples were prepared as described above for live-cell microscopy. In order to ensure a high labeling density with exchangeable fluorophore ligand,[33,34] a concentration of 100 nM SiR-S5 and JF_{585}-Hy4 in the imaging buffer was used. Confocal microscopy experiments were conducted at room temperature on a commercial SP8 confocal laser scanning microscope (Leica Microsystems, Wetzlar, Germany) equipped with an HC PL APO CS2 20x/0.75 immersion objective (Leica Microsystems). Images were acquired with the Leica Application Suite X Software (v3.5.7.23225, Leica Microsystems). 16-bit images with a size of 1024×1024 px at 757.58 nm pixel size were acquired with a scan speed of 400 Hz and a line average of 2. SiR was excited with a 633 nm HeNe laser at 0.2% intensity and its emission was detected with a HyD detector (gain 160) in a 638-784 nm detection window. JF_{585} was excited with a 561 nm HeNe laser at 0.5% intensity and its emission was detected with a HyD detector (gain 160) in a 573-628 nm detection window.

Data analysis

SPT data were analyzed using a pipeline for single-particle tracking analysis as described in detail elsewhere.[12,13,41] In brief, raw data were localized using ThunderSTORM (dev-2016-09-10-b1),[64] a plugin for Fiji,[65] localizations were connected to trajectories in swift (v0.4.3),[66] and parameter estimation for swift as well as the diffusion analysis was conducted in SPTAnalyser (v.1.2.0).[41] Parameters were determined according to the SPTAnalyser software manual (v1.2.0) and chosen as published previously,[13] if not stated otherwise in the following. For SiR-labeled ligands, the parameter *diffraction_limit* was set to 18 nm, *exp_displacement* to 125 nm, *p_bleach* to 0.025, and D_{min} to 0.0041 μm^2/s. For JF_{585}-labeled ligands, *diffraction_limit* = 15 nm, *exp_displacement* = 135 nm, *p_bleach* = 0.08 nm, and D_{min} = 0.0057 μm^2/s was applied.
SPTAnalyser including scripts for batch processing is available on github (https://github.com/HeilemannLab/SPTAnalyser) together with detailed documentation.
For long-term measurements, the localizations per frame were extracted from the localization lists, normalized to the area of the cell, binned into 1 min (3,000 frames) intervals, and normalized to the first frame. The diffusion coefficients per frame were directly extracted from the output files from swift and filtered for mobility type and trajectory length. Only diffusion coefficients of trajectories classified as mobile and longer than 20 frames were kept. For every trajectory, only a single diffusion coefficient was counted in the frame in which the respective trajectory appeared. To visualize the activation profile of EGFR, the diffusion coefficients per frame of EGF-treated cells were binned into 1 min intervals and subtracted by the diffusion coefficients per frame of resting cells, to yield the difference in diffusion coefficients ΔD.
All errors represent the standard error of the mean (SEM). OriginPro 2024 (v10.1.0.170, OriginLab Corporation, Northampton, MA, USA) was used for statistical analysis. The Shapiro-Wilk test (α = 0.05) was applied to test populations for normality. The Mann-Whitney U test was applied for statistical testing as some data sets rejected normality. p-values < 0.001 are marked as highly significantly different (***).

Supporting Figures

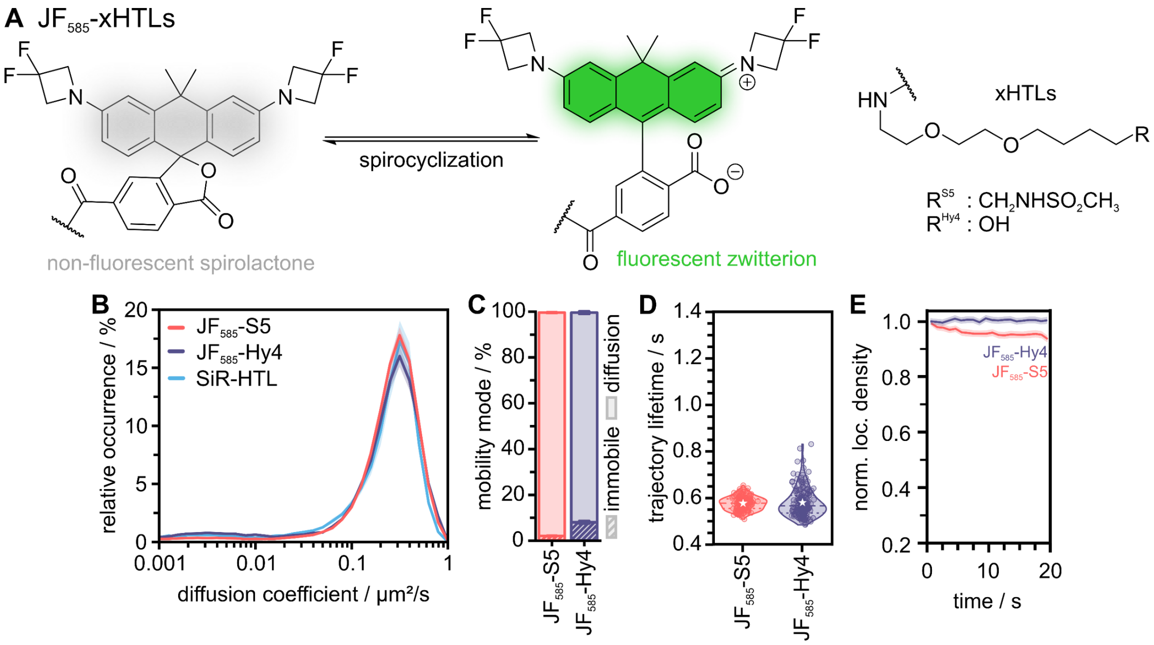

Figure S1: CD86-HT7 and CD86-dHT7 imaged with the xHTLs JF_{585}-S5 and JF_{585}-Hy4.

(A) Chemical structures of the reversible equilibrium between the non-fluorescent spirocyclic state and fluorescent zwitterionic state of JF_{585} and different JF_{585}-derivatives of xHTLs.

(B) Relative occurrence of the mean diffusion coefficient per cell for JF_{585}-tagged exchangeable HaloTag Ligands.

(C) Percentage of mobility modes per cell for JF_{585}-S5 and JF_{585}-Hy4. Single-molecule trajectories were assigned to the classes immobile or diffusion.

(D) Lifetime of single-molecule trajectories of the non-covalent JF_{585}-S5 binding to the HaloTag7 and JF_{585}-Hy4 binding to the dead mutant of the HaloTag7. Dashed lines represent the median, stars the mean, and dotted lines the interquartile range.

(E) Mean number of localizations per area binned into 1 s intervals, normalized to the respective data in the first frame and plotted over time for JF_{585}-S5 (red) and JF_{585}-Hy4 (purple).

All errors represent the SEM (N = 180 cells for all conditions).

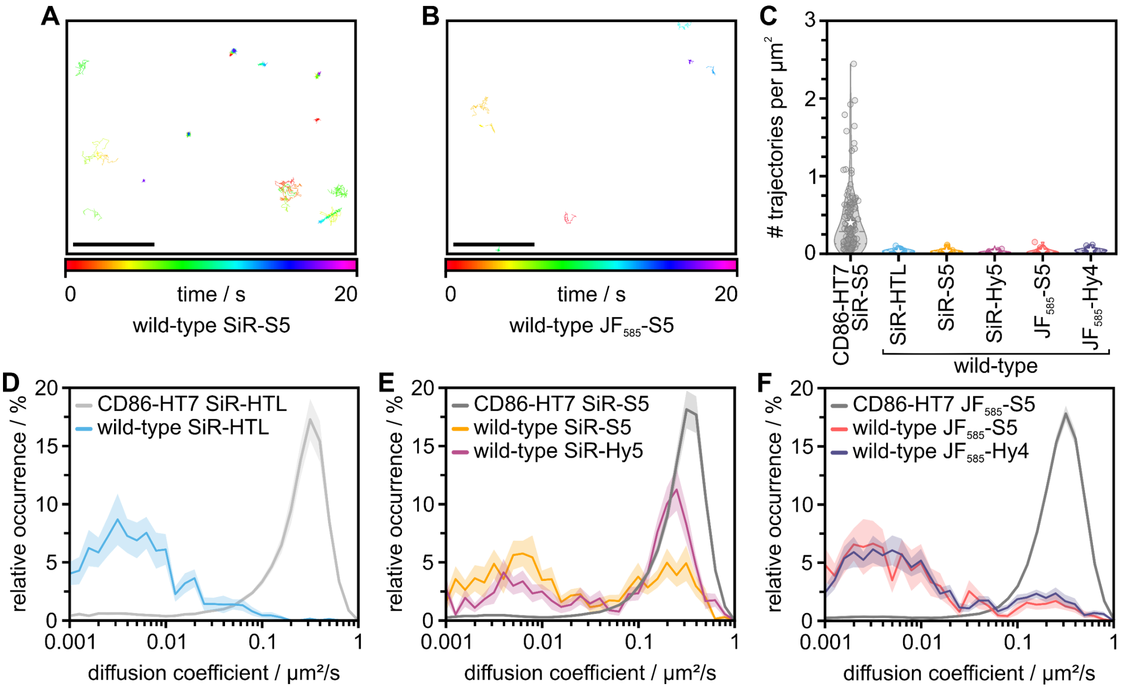

Figure S2: U-2 OS wild-type cells imaged with covalent and exchangeable HTLs as negative controls.
Single-molecule trajectories of exemplary cells acquired using the (A) SiR-S5 and (B) JF_{585}-S5. Trajectories are color-coded over the 20 s acquisition time. Scale bar 5 µm.
(C) Number of trajectories per area for each ligand in U-2 OS wild-type cells compared to CD86-HT7 imaged with SiR-S5. Very few trajectories were detected in the negative controls. Dashed lines represent the median, stars the mean, and dotted lines the interquartile range.
(D-F) Relative occurrence of the mean diffusion coefficient per cell for each ligand in U-2 OS wild-type cells compared to the respective CD86-HT7 cell line.
All errors represent the SEM (N = 20 cells for negative controls, N = 160 cells for CD86-HT7 imaged with SiR-S5 and SiR-HTL, N = 180 cells for CD86-HT7 imaged with JF_{585}-S5).

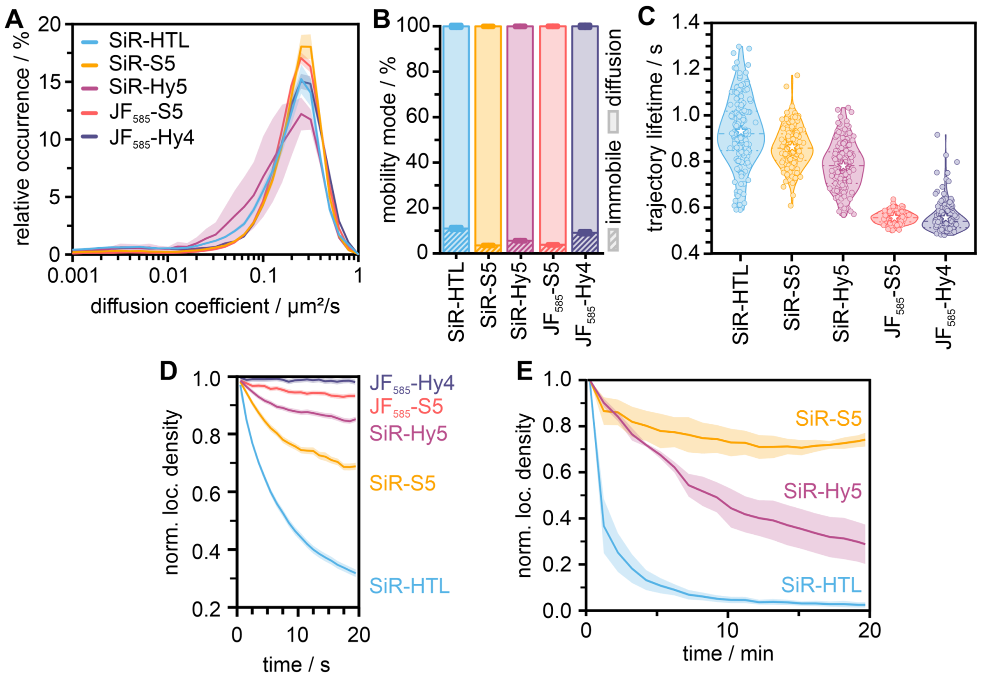

Figure S3: CTLA-4-HT7 and CTLA-4-dHT7 imaged with the covalent HaloTag Ligand SiR-HTL as well as the xHTLs SiR-S5, SiR-Hy5, JF_{585}-S5, and JF_{585}-Hy4.

(A) Relative occurrence of the mean diffusion coefficient per cell for SiR-HTL (blue), SiR-S5 (yellow), SiR-Hy5 (magenta), JF585-S5 (red), and JF585-Hy4 (purple).

(B) Percentage of mobility modes per cell for the different ligands. Single-molecule trajectories were assigned to the classes immobile or diffusion.

(C) Lifetime of single-molecule trajectories of the different ligands. Dashed lines represent the median, stars the mean, and dotted lines the interquartile range.

(D) Mean number of localizations per area from short SPT measurements binned into 1 s intervals, normalized to the respective data in the first frame, and plotted over time for the different ligands.

(E) Mean number of localizations per area from long-time SPT measurements binned into 1 min intervals, normalized to the respective data in the first frame, and plotted over time for SiR-HTL (blue), SiR-S5 (yellow), and SiR-Hy5 (magenta).

All errors represent the SEM (N = 160 cells for SiR-tagged ligands and N = 180 cells for JF_{585}-tagged ligands in A-D, and N = 4 cells for E)

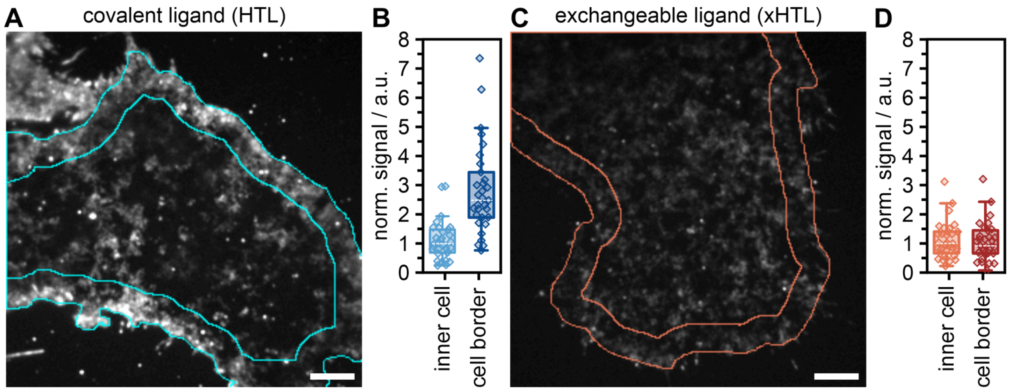

Figure S4: Comparison of the fluorescence signal in cells imaged with the covalent ligand SiR-HTL (A, B) and the exchangeable probe SiR-S5 (C, D). For the analysis, a full 20 s SPT measurement was z-projected with the setting "standard deviation" in Fiji (A, C) and the median intensity value measured either in the region of the border or inside of the cell (B, D). The median intensity values per cell were normalized to the overall median intensity inside the cells per condition. 31 cells were analyzed for each condition. The box plots display the median (dashed white line) with the 25th and 75th percentile with whiskers reaching to the last data point within the 1.5× interquartile range, and diamonds represent single-cell values.

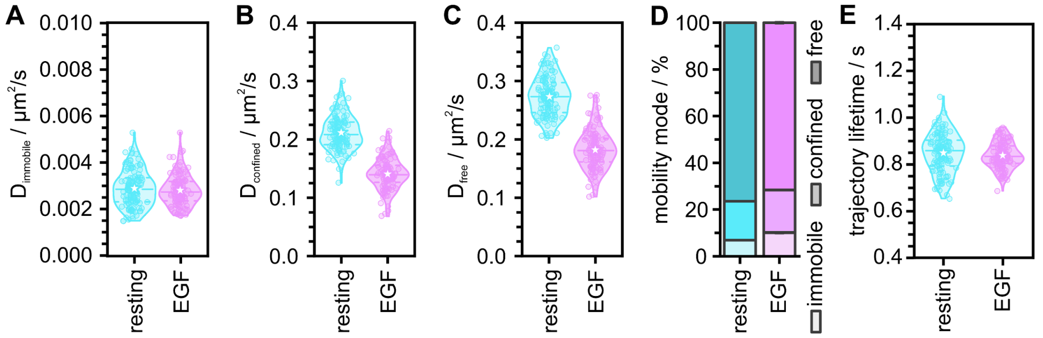

Figure S5: Diffusion properties of EGFR-HT7 in resting and EGF-stimulated U-2 OS cells. SiR-S5 was used as xHTL.

(A-C) Distribution of diffusion coefficients for the individual diffusion modes immobile (A), confined (B), and free (C). Dashed lines represent the median, dotted lines represent the quartiles, and stars represent the mean. In the data seen in **Figure 3**, the confined and freely moving receptor fractions were pooled.

(D) Occurrence of immobile molecules and confined and freely diffusing EGFR-HT7.

(E) Lifetime of single-molecule trajectories extracted from SPT data of EGFR-HT7. Dashed lines represent the median, stars the mean, and dotted lines the interquartile range.

160 cells were analyzed per condition for all plots.

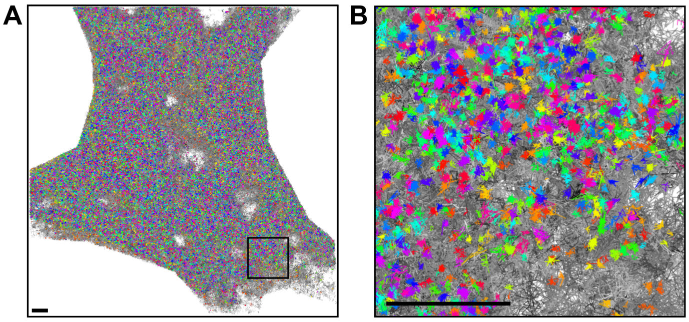

Figure S6: Related to **Figure 3FG**.

(A) Overlay of all trajectories (gray) and only immobile trajectories (rainbow, randomly color-coded) throughout a 30 min measurement of EGFR-HT7.

(B) Zoom-in of the region marked in (A). Scale bars 2 µm.

Supporting Tables

Supplementary Table S1: Mean diffusion coefficients of all measured protein-ligand combinations calculated from 20 s measurements. The diffusion coefficient of mobile particles (D_{mobile}) is calculated as the mean value of the diffusion coefficient of the confined ($D_{confined}$) and free (D_{free}) mobilities modes weighted by their relative occurrence (**Table S2**). The global diffusion coefficient D_{global} is calculated similarly including all three mobility types (immobile, confined, free). POI: protein of interest. All given errors are the SEM.

POI	ligand	D_{global} / µm²s⁻¹	$D_{immobile}$ / µm²s⁻¹	D_{mobile} / µm²s⁻¹	$D_{confined}$ / µm²s⁻¹	D_{free} / µm²s⁻¹
CD86-HT7	SiR-HTL	0.294 ± 0.007	0.0043 ± 0.0009	0.315 ± 0.007	0.245 ± 0.016	0.336 ± 0.007
CD86-HT7	SiR-S5	0.300 ± 0.011	0.0036 ± 0.0010	0.319 ± 0.010	0.262 ± 0.03	0.331 ± 0.011
CD86-HT7	JF_{585}-S5	0.333 ± 0.008	0.0046 ± 0.0013	0.344 ± 0.008	0.296 ± 0.016	0.360 ± 0.009
CD86-dHT7	SiR-Hy5	0.283 ± 0.005	0.0034 ± 0.0006	0.300 ± 0.005	0.245 ± 0.013	0.312 ± 0.006
CD86-dHT7	JF_{585}-Hy4	0.31 ± 0.02	0.0044 ± 0.0017	0.34 ± 0.02	0.29 ± 0.04	0.36 ± 0.02
CTLA-4-HT7	SiR-HTL	0.228 ± 0.008	0.0051 ± 0.0011	0.255 ± 0.008	0.194 ± 0.017	0.274 ± 0.009
CTLA-4-HT7	SiR-S5	0.270 ± 0.005	0.0042 ± 0.0011	0.279 ± 0.005	0.235± 0.012	0.288 ± 0.005
CTLA-4-HT7	JF_{585}-S5	0.285 ± 0.005	0.007 ± 0.002	0.291 ± 0.005	0.249 ± 0.009	0.303 ± 0.006
CTLA-4-dHT7	SiR-Hy5	0.257 ± 0.004	0.0041 ± 0.0006	0.271 ± 0.004	0.222 ± 0.009	0.283 ± 0.004
CTLA-4-dHT7	JF_{585}-Hy4	0.272 ± 0.015	0.006 ± 0.002	0.295 ± 0.016	0.248 ± 0.03	0.311 ± 0.018
EGFR-HT7 rest.	SiR-S5	0.245 ± 0.004	0.0029 ± 0.0005	0.262 ± 0.004	0.211 ± 0.010	0.273 ± 0.004
EGFR-HT7 +EGF	SiR-S5	0.158 ± 0.003	0.0028 ± 0.0004	0.174 ± 0.003	0.140 ± 0.007	0.182 ± 0.004

Supplementary Table S2: Mean relative occurrence of mobility modes of all measured protein-ligand combinations calculated from 20 s measurements. The mobile fraction is the sum of the confined and free mobility modes. All given errors are the SEM.

POI	ligand	immobile / %	mobile / %	confined / %	free / %
CD86-HT7	SiR-HTL	7.6 ± 0.3	92.4 ± 0.3	18.4 ± 0.3	74.0 ± 0.4
CD86-HT7	SiR-S5	6.1 ± 0.3	93.4 ± 0.4	16.8 ± 0.2	77.1 ± 0.3
CD86-HT7	JF_{585}-S5	3.6 ± 0.2	98.07 ± 0.15	22.5 ± 0.2	73.9 ± 0.3
CD86-dHT7	SiR-Hy5	6.6 ± 0.4	93.9 ± 0.3	16.0 ± 0.3	77.4 ± 0.4
CD86-dHT7	JF_{585}-Hy4	9.0 ± 0.6	92.0 ± 0.6	22.5 ± 0.4	68.5 ± 0.7
CTLA-4-HT7	SiR-HTL	10.8 ± 0.6	89.2 ± 0.6	20.5 ± 0.3	68.6 ± 0.7
CTLA-4-HT7	SiR-S5	3.4 ± 0.2	96.6 ± 0.2	16.9 ± 0.2	79.8 ± 0.3
CTLA-4-HT7	JF_{585}-S5	1.9 ± 0.2	96.4 ± 0.2	23.9 ± 0.2	74.1 ± 0.2
CTLA-4-dHT7	SiR-Hy5	5.4 ± 0.2	94.6 ± 0.2	18.9 ± 0.2	75.7 ± 0.3
CTLA-4-dHT7	JF_{585}-Hy4	8.0 ± 0.6	91.0 ± 0.6	23.9 ± 0.3	68.1 ± 0.6
EGFR-HT7 rest.	SiR-S5	6.9 ± 0.2	93.1 ± 0.2	16.8 ± 0.2	76.3 ± 0.2
EGFR-HT7 ±EGF	SiR-S5	10.1 ± 0.4	89.9 ± 0.4	17.50 ± 0.14	72.44 ± 0.4

Supplementary Table S3: Mean trajectory lifetime of all measured protein-ligand combinations given in seconds calculated from 20 s measurements. The mean value of the global lifetime is calculated as the mean value of the values extracted from trajectories classified as immobile, confined, and freely diffusing weighted by their relative occurrence (**Table S2**). All given errors are the SEM.

POI	ligand	trajectory life-time global / s	trajectory life-time immobile / s	trajectory life-time confined / s	trajectory life-time free / s
CD86-HT7	SiR-HTL	0.94 ± 0.03	0.88 ± 0.10	0.63 ± 0.03	1.02 ± 0.04
CD86-HT7	SiR-S5	0.93 ± 0.04	0.85 ± 0.16	0.64 ± 0.04	0.99 ± 0.05
CD86-HT7	JF_{585}-S5	0.576 ± 0.010	0.68 ± 0.10	0.522 ± 0.013	0.586 ± 0.012
CD86-dHT7	SiR-Hy5	0.87 ± 0.02	0.83 ± 0.09	0.62 ± 0.02	0.92 ± 0.02
CD86-dHT7	JF_{585}-Hy4	0.58 ± 0.03	0.76 ± 0.18	0.51 ± 0.03	0.57 ± 0.03
CTLA-4-HT7	SiR-HTL	0.93 ± 0.04	0.89 ± 0.12	0.62 ± 0.03	1.02 ± 0.05
CTLA-4-HT7	SiR-S5	0.86 ± 0.02	0.75 ± 0.09	0.62 ± 0.02	0.91 ± 0.02
CTLA-4-HT7	JF_{585}-S5	0.556 ± 0.006	0.60 ± 0.07	0.513 ± 0.009	0.567 ± 0.008
CTLA-4-dHT7	SiR-Hy5	0.780 ± 0.012	0.74 ± 0.05	0.600 ± 0.014	0.825 ± 0.015
CTLA-4-dHT7	JF_{585}-Hy4	0.56 ± 0.02	0.71 ± 0.13	0.50 ± 0.02	0.55 ± 0.03
EGFR-HT7 rest.	SiR-S5	0.85 ± 0.02	0.76 ± 0.05	0.63 ± 0.02	0.90 ± 0.02
EGFR-HT7 ±EGF	SiR-S5	0.85 ± 0.02	0.75 ± 0.04	0.63 ± 0.02	0.90 ± 0.02

Supplementary Table S1: Mean diffusion coefficients and mean relative occurrences of mobility modes of CalR-HT7-KDEL. The diffusion coefficient of restricted particles ($D_{restricted}$) is calculated as the mean value over the diffusion coefficients assigned to each trajectory, classified as confined ($D_{confined}$) or immobile ($D_{immobile}$) during the diffusion analysis. The restricted fraction is the sum of the confined and immobile mobility modes. The mobile fraction corresponds to the freely moving particles. POI: protein of interest. All given errors are the SEM.

POI	ligand	$D_{restricted}$ / µm²s⁻¹	D_{mobile} / µm²s⁻¹	restricted / %	mobile / %
CalR-HT7-KDEL	SiR-HTL	0.14 ± 0.05	0.39 ± 0.04	49 ± 2	51 ± 2
CalR-HT7-KDEL	SiR-S5	0.14 ± 0.04	0.36 ± 0.04	58 ± 8	42 ± 8

References

[59] F. Fricke, J. Beaudouin, R. Eils, M. Heilemann, *Sci. Rep.* **2015**, *5*, 14072.
[60] R. E. Carter, A. Sorkin, *J. Biol. Chem.* **1998**, *273*, 35000–35007.
[61] D. G. Gibson, L. Young, R.-Y. Chuang, J. C. Venter, C. A. Hutchison 3rd, H. O. Smith, *Nat. Methods* **2009**, *6*, 343–345.
[62] M. J. Malecki, C. Sanchez-Irizarry, J. L. Mitchell, G. Histen, M. L. Xu, J. C. Aster, S. C. Blacklow, *Mol. Cell. Biol.* **2006**, *26*, 4642–4651.
[63] A. Edelstein, N. Amodaj, K. Hoover, R. Vale, N. Stuurman, *Curr. Protoc. Mol. Biol.* **2010**, *Chapter 14*, Unit14.20.
[64] M. Ovesný, P. Křížek, J. Borkovec, Z. Svindrych, G. M. Hagen, *Bioinformatics* **2014**, *30*, 2389–2390.
[65] J. Schindelin, I. Arganda-Carreras, E. Frise, V. Kaynig, M. Longair, T. Pietzsch, S. Preibisch, C. Rueden, S. Saalfeld, B. Schmid, J.-Y. Tinevez, D. J. White, V. Hartenstein, K. Eliceiri, P. Tomancak, A. Cardona, *Nat. Methods* **2012**, *9*, 676–682.
[66] M. Endesfelder, C. Schießl, B. Turkowyd, T. Lechner, U. Endesfelder, manuscript in preparation.
